# The complex of miRNA2861 and cell-penetrating, dimeric α-helical peptide accelerates the osteogenesis of mesenchymal stem cells

**DOI:** 10.1186/s40824-022-00336-9

**Published:** 2022-12-29

**Authors:** So Hee Nam, Yan Lee, Chi-Heon Kim, Dong Eun Kim, Hee-Jin Yang, Sung Bae Park

**Affiliations:** 1 College of Pharmacy, Dongduk Women’s University, Seoul, Korea; 2 Department of Chemistry, Seoul National University, Seoul, Korea; 3 Department of Neurosurgery, Seoul National University College of Medicine, Seoul, Korea; 4 Clinical Research Institute, Seoul National University Hospital, Seoul, Korea; 5 Department of Neurosurgery, Seoul National University Boramae Medical Center, 20 Boramae-Ro 5-Gil, Dongjak-Gu, Seoul, 07061 Korea

**Keywords:** Osteogenesis, miRNA, Mesenchymal stem cell, Cell penetrating peptide, Osteoporosis

## Abstract

**Background:**

The restoration of the functional ability of mesenchymal stem cells (MSCs) using epigenetic modification is very promising for patients with weak osteogenesis ability. This study focused on the acceleration of osteogenesis from MSCs using microRNA (miRNA)2861 and a cell-penetrating peptide (CPP), LK.

**Methods:**

We performed MSCs penetration test of complex between the LK peptides and miRNA 2861. Three different experiments were performed to investigate the effects of miRNA 2861 on osteogenic differentiation in MSCs: 1) intensity of alizarin red staining, which reflects the status of mineralization by osteoblasts; 2) gene expression related to osteoblast differentiation; and 3) confirmation of corresponding protein translation for comparison with RNA expression levels.

**Results:**

We found that cLK effectively delivered miRNA 2861 into the cytoplasm of human MSCs and accelerated osteogenic differentiation from MSCs, as well as mineralization.

**Conclusion:**

The complex of miRNA 2861 with LK may have a positive effect on the osteogenic differentiation from MSCs and mineralization. Therapies using miRNAs combined with LK may be good candidates for the augmentation of osteogenesis in patients.

**Supplementary Information:**

The online version contains supplementary material available at 10.1186/s40824-022-00336-9.

## Introduction

Although the increase in life expectancy and the improvement of surgical techniques have allowed elderly patients to maintain or recover an active physical condition, the number of elderly patients who need surgical treatment has been increasing [1]. However, the aging population with or without osteoporosis is likely to have weak bone strength and impaired capacity of bone repair because of negative bone remodeling [2, 3]. Therefore, alternative strategies for bone repair and formation may be particularly considered in the aging population. Among those strategies, we may consider adequate medication for osteoporosis and underlying diseases of the patients at the perioperative period and effective bone grafting or replacement to enhance bone formation at the operative field.

Regarding bone grafting, because auto-bone grafts (from the patient’s own bone) can provide an osteoconductive scaffold, osteoinductive factors, and osteogenic cells into the bone-remodeling site, the use of autologous bone remains the gold-standard approach for bone repair or formation in the surgical field [4, 5]. However, autologous bone grafting is associated with various perioperative and postoperative complications, such as morbidity on the donor site, limited bone supply, and variability in the osteogenic ability depending on the patient’s age and medical history, including osteoporosis [5, 6]. Therefore, bone-substitute development is warranted according to factors specific to each patient, including age, sex, and underlying diseases.

With increasing age, the bone resorption activity is increased and the functional potency of mesenchymal stem cells (MSCs) into osteocytes is decreased, whereas the percentage of adipocytes is increased. Therefore, the self-renewal capacity, i.e., osteogenesis, decreases gradually [7]. Because of the complications mentioned above and the decreased capacity for osteogenesis, there is a need for the development of other bone substitutes to enhance bone healing and positive bone remodeling. Biomimetic materials, the bone morphogenetic protein (BMP), extracellular matrix mimicry materials, polymer scaffolds, MSC-based therapies, and nanomaterials have been considered as alternatives [8]. In particular, in the case of elderly patients with weak osteogenesis ability, bone substitutes can restore the functional ability of MSCs from adipogenesis into osteogenesis. Among the methods that can increase osteogenesis, cell-intrinsic factors, such as epigenetic modification, have been reported to be more efficient in osteogenesis by directly affecting the differentiation of MSCs into osteoblast without co-stimulation of other intracellular pathways involved in adipogenesis [9].

Several studies have reported that histone acetylase inhibitors (HDACs) and micro RNAs (miRNAs) can act on transcription as activators or attenuators of histone modification during the epigenetic modifications associated with osteoblast differentiation [10, 11]. miRNAs are endogenous non-coding RNAs that comprise a small number of nucleotides (~ 25) and can regulate gene expression by degrading mRNAs or inhibiting mRNA translation [12]. Several miRNAs have been reported to regulate the translation of HDACs. miRNAs 188, 22, 29b, 2861, and 449a inhibit the translation of HDAC1, HDAC4, HDAC5, and HDAC6 and promote osteogenesis [12–14].

Among various target miRNA candidates, here we investigated the ability of miRNA 2861 to enhance osteogenesis on MSCs through the inhibition of the translation of HDAC5 [15]. Previously, we reported that cell-penetrating peptides (CPPs) can deliver the Runx2 protein, and that the complex of CPP with Runx2 augmented osteogenesis on MSCs [16]. A cyclic α-helical CPP (i.e., LK) based on leucine and lysine residues (cLK) efficiently delivered the transcription factor Runx2 into MSCs. Similarly, in this study, we investigated whether miRNA 2861 and the CPP complex can augment osteoblast differentiation and whether the complex enhances the modulation of the known target, HDAC5, using human bone-marrow-derived MSCs [15].

## Experimental section (Materials and methods)

### Cultivation of human bone-marrow-derived MSCs

We transferred human MSCs (BM027SS15-P2) from the Catholic Institute of Cell Therapy, Catholic Medical Center, for use in the present study. The study protocol was approved by the Use Committee of the Clinical Research Institute, Seoul National University Hospital, Seoul, Korea (permit number: 07–2020-6). These cells were maintained in DMEM-Low Glucose (Biowest, Missouri, USA) containing 20% fetal bovine serum (Biowest, Missouri, United States) at 37 °C with an atmosphere of 5% CO_2_. The medium was changed every 3–4 days. To expand the cells, the adhered monolayer was detached using trypsin/EDTA (Gibco, ThermoFisher, Massachusetts, USA) for 5 min at 37 °C.

### Materials for peptide synthesis

N-α-Fmoc–protected amino acids, Rink Amide MBHA resin (0.078 mmol/g loading), and benzotriazole-1-yl-oxy-tris-pyrrolidino-phosphonium hexafluorophosphate (PYBOP) were purchased from Novabiochem (San Diego, CA, USA). Dimethylformamide (DMF), 1,2-dichloromethane (DCM), N,N-diisopropylethylamine (DIPEA), trifluoroacetic acid (TFA), triisopropylsilane (TIS), triethylsilane (TES), piperidine, and 5(6)-carboxytetramethylrhodamine (TAMRA) were purchased from Sigma-Aldrich (St. Louis, MO, USA). n-Hexane and diethyl ether were purchased from Daejung (Siheung, Gyeonggi, Korea).

### Synthesis and characterization of LK peptides

**Fig. 1:**
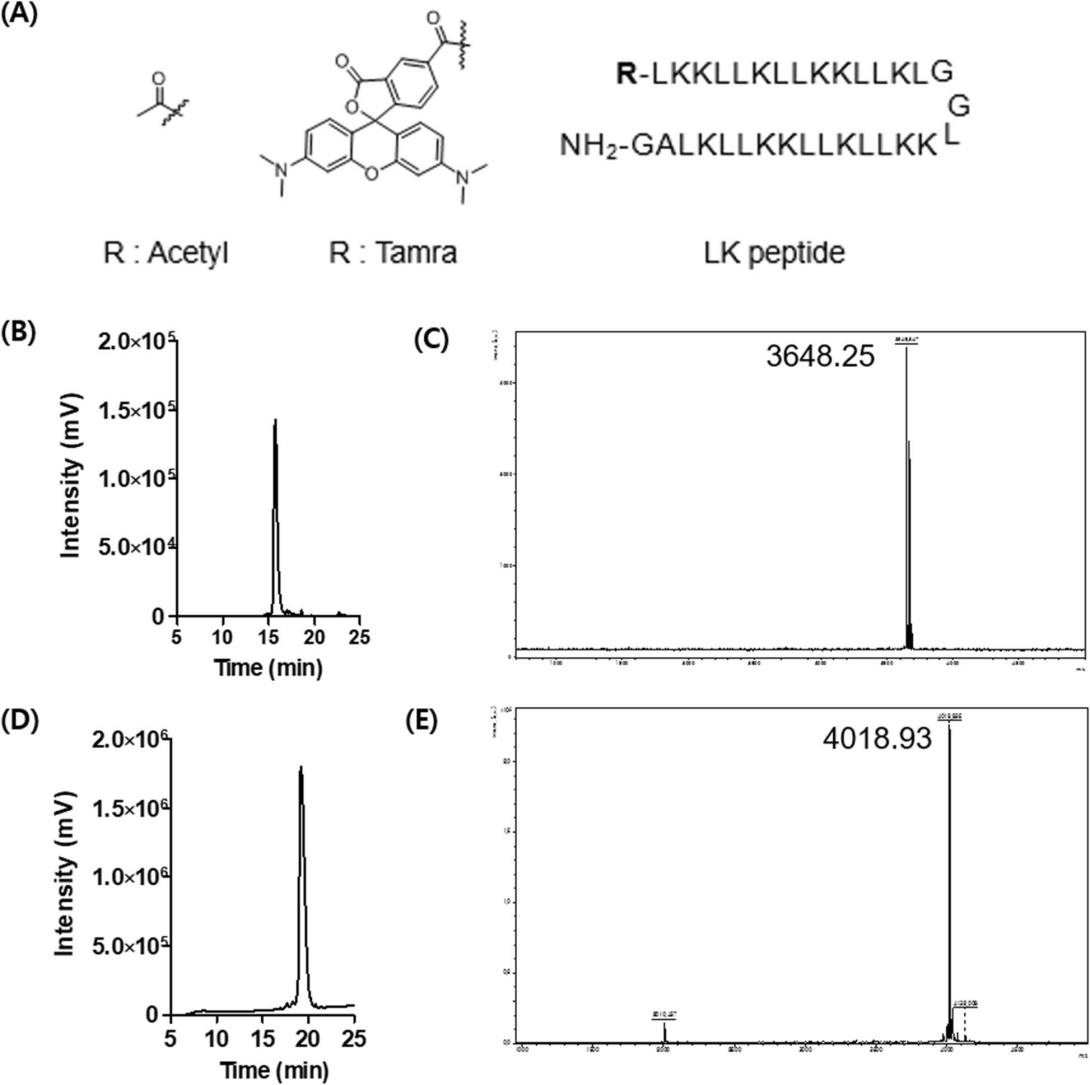
Characterizations of ac-LK and Tamra-LK. **A** Chemical structures and amino acid sequences of acetylated and Tamra-labeled LK. **B**-**E** The HPLC chromatograms and MALDI-TOF mass spectras of ac-LK (**B**, **C**) and Tamra-labeled LK (**D**, **E**). Both CPPs were analyzed by HPLC with 70–75% of ACN (0.1% TFA) (> 97% purity) at 220 nm. Ac-LK; MS [M + H]^+^ 3648.62 (calcd), 3648.25 (found), Tamra-LK; MS [M + H].^+^ 4018.75 (calcd), 4018.93 (found)

We prepared all CPPs via Fmoc-based solid-phase peptide synthesis using Rink Amide MBHA resin (0.07 mmol scale) [17]. First, Rink Amide MBHA resin was deprotected using 20% piperidine in DMF. *N*-α-Fmoc–protected amino acids were conjugated to the resin using PYPOB (0.42 mmol) and DIPEA (0.42 mmol) in DMF, and deprotected using 20% piperidine in DMF. Subsequently, the coupling of amino acids and Fmoc deprotection steps were repeated sequentially until the last amino acid using a microwave peptide synthesizer (CEM, Matthews, NC, USA) with the irradiation set at 5 W for 5 min. For the acetylation of the LK peptide, 0.03 mmol of the resin-bound peptide was added to a solution of acetic anhydride (0.18 mmol) and HOBt (0.18 mmol) in 2 mL of DMF:DCM (v/v) = 90:10. For the synthesis of the TAMRA-conjugated LK peptide (TAMRA-LK), 0.02 mmol of the resin-bound peptide was reacted with a 1.3-fold molar excess of 5(6)-TAMRA (0.26 mmol), PYBOP (0.026 mmol), and DIPEA (0.12 mmol) in 2 mL of DMF for 2 h. The peptides were cleaved from the resin by treating them with a mixture of 2 mL TFA/TIS/water (v/v) = 96.5:1:2.5 for 2 h. After the cleavage, peptides were precipitated with diethyl ether and lyophilized. Finally, the peptide was purified with HPLC (Agilent 1100 series) with an Agilent C18 (3.5 mm, 4.6 × 150 mm) column. Solution A (water with 0.1% v/v TFA) and Solution B (ACN with 0.1% v/v TFA) were used as the eluent solutions. The HPLC chromatogram and MALDI-TOF mass spectra of purified LK peptides are shown in Fig. 1 (> 97% purity): ac-LK; MS [M + H]^+^: 3648.62 (calcd.), 3648.25 (found) TAMRA-LK; MS [M + H]^+^: 4018.75 (calcd.), 4018.93 (found).

### Gel shift assay

miRNA 2861 (Sigma-Aldrich) and ac-LK were prepared in 0.5 × PBS at various molar equivalent ratios and incubated for 1 h. Next, miRNA 2861 only and complexes were analyzed by electrophoresis on 1% agarose gels, followed by staining with ethidium bromide and gel imaging using the Gel Doc imaging system (Bio-Rad Laboratories, Korea).

### Dynamic Light Scattering (DLS) analysis

The formation of a Complex A, B and C between ac-LK and miRNA 2861 was examined by DLS using a Zetasizer instrument (Malvern, Zetasizer Nano ZS). The solutions of the ac-LK and miRNA 2861 were prepared as a 2X stock solution in 100 mM Tris buffer. The same volume of the two solutions was mixed and incubated for 30 min before measurements.

### Transmission electron microscopy (TEM) analysis

The structure of complex of LK and miRNA was examined by the electron-filtering transmission electron microscopy (TEM, Carl Zeiss, LIBRA 120) with accelerating voltage of 120 kV. The complex was prepared at a concentration of 2 μM of miRNA and fRunx2 with 6 and 12 equivalent amount of ac-LK in deionized water. Samples were loaded on the carbon-coated copper grid (200 mesh) and stained with a 2% uranyl acetate solution and analyzed.

### In vitro cytotoxicity test

MSCs were seeded in 96-well tissue culture dishes (3 × 10^3^ cells per well) and incubated overnight at 37 °C. Next, the cells were cultured in fresh medium containing ac-LK at various concentrations (10 nM to 1 μM) for 48 h at 37 °C. Subsequently, the cells were washed three times with PBS and fresh medium containing10 μL of a CCK solution (Dojindo, Kumamoto, Japan) was added. After incubation for 1 h at 37 °C, the absorbance was measured at 430 nm using a microplate reader (Molecular Devices, Menlo Park, CA, USA), and the relative cell viability was calculated by comparison with untreated control cells.

### In vitro penetration test

MSCs (2 × 10^4^ cells per well) were seeded in 24-well plates and incubated overnight at 37 °C. TAMRA-LK was added to the cells at various concentrations (50 − 1,000 nM). To investigate the cell-penetration activity of miRNA 2861 with ac-LK, preincubated complexes of Fam-labeled miRNA 2861 and ac-LK were administered to the cells at various concentrations. The complexes were prepared using 50 nM miRNA 2861 and 1, 3, and 6 molar equivalent amounts of LK. In addition, the complex of 50 nM PrEST Antigen Runx2 (fRunx2, Sigma-Aldrich) and 500 nM ac-LK was added together with the complex of miRNA 2861 and ac-LK (6 equiv.). Lipofectamine™ 2000 (Thermo Fisher) was used as a control transfection reagent and the complex was prepared by adding 1 μL of lipofectamine based on 5 pmol of miRNA according to the instruction manual. After a 24-h incubation, the cells were washed three times with PBS and incubated with trypsin–EDTA for 5 min. The detached cells were centrifuged at 1,200 rpm for 3 min and suspended in 300 μL of PBS containing 2% FBS. The degree of cellular uptake was analyzed using a flow cytometer (BD Accuri C6, BD Biosciences). The experiments were performed three times for each group and the data were presented as fluorescence-positive cells (%) compared with the control cells with no fluorescence.

Moreover, confocal laser scanning microscopy (CLSM, Zeiss, Germany) was used for the observation of the fluorescence images. Cells were seeded at a density of 10,000 cells/dish on 35-mm glass-bottom dishes (SPL Lifescience) and incubated for 24 h. The culture medium was exchanged with 180 μL of a fresh medium with 10% FBS, and 20 μL of a 10 × stock solution of each complex was added to the medium. After incubation for 24 h, the cells were washed with fresh medium and stained with a Hoechst 33,342 solution (4 μM) for 15 min. For investigating the endosomal escape, cells were stained additionally with 500 nM of Lysotracker™ Deep Red (Thermo Fisher) for 30 min. Then, the cells were washed three times with PBS and fresh medium was added. CLSM images were acquired using a Zeiss DE/LSM 510 NLO microscope (Carl Zeiss, Germany) with a 500 × objective (CApochromat, Carl Zeiss).

### Osteoblast differentiation

Three different experiments were performed to investigate the effects of miRNA 2861 on osteogenic differentiation in MSCs: 1) intensity of alizarin red staining, which reflects the status of mineralization by osteoblasts; 2) gene expression related to osteoblast differentiation; and 3) confirmation of corresponding protein translation for comparison with RNA expression levels.

MSCs from passage 5 were seeded overnight in DMEM-low medium without FBS and antibiotics at a density of 100 cells/mm^2^ in a 6-well culture plate. After overnight incubation, the medium was replaced with Opti-MEM (Gibco, Thermo Fisher, Massachusetts, United States). Cells were then divided into six groups and treated with PBS (as a control), miRNA 2861 only, ac-LK only, Complex A (50 nM miRNA 2861 and 300 nM ac-LK), Complex B (50 nM miRNA 2861 and 600 nM ac-LK), and Complex C (Complex B and the complex of 50 nM fRunx2 and 500 nM ac-LK). The role of fRunx2 was augmentation of osteoblast differentiation from MSCs. One day later, the cells were washed with PBS and incubated continuously in osteogenic medium (OM) (StemPro® Osteogenesis Differentiation Kit; Gibco, New York, NY, USA). Mineralization was measured at 7, 14, and 21 days.

The progression of mineralization, which was confirmed by alizarin red staining, was used to assess osteoblast differentiation. Briefly, cells were washed with PBS and fixed with 70% ethanol for 1 h at 4 °C. After washing with deionized water, cells were stained with fresh 2% alizarin red S solution (pH 4.2) for 30 min and washed five times with deionized water. The mineralization of cells was examined by microscopy. For the quantification of alizarin red staining, the stained cells were incubated in 10% (w/v) cetylpyridinium chloride in 10 mM sodium phosphate for 1 h at room temperature, to extract the alizarin red. Two hundred microliters of media was moved to 96-well plates and analyzed by spectrophotometry at 562 nm. All experimental groups were triplicated.

### Gene expression

After treatment with miRNA only and the complexes, the cells were harvested and total RNA was isolated using the TRIzol reagent (Ambion, Life Technologies, Carlbad CA, USA) at days 7, 14, and 21, according to the manufacturer’s instructions. One microgram of RNA was reverse transcribed into cDNA with the AccuPower® Rocket Script RT PreMix & MasterMix (Bioneer, Daejeon, South Korea). qRT-PCR was performed in 7 μL of water with 1 μL of primer (10 pmol) and 10 μL of qPCR Master Mix (Bioneer, Daejeon, South Korea); the products were analyzed on a Real-Time PCR Instrument System (Light Cycler 480, Roche). The initial pre-denaturation of cDNA was achieved at 95 °C for 15 s, followed by 40 cycles of denaturation at 95 °C for 1 min, 60 °C for 30 s, and annealing and extension at 60 °C for 1 min/cycle. Amplified products were measured continuously by fluorescence emission.

The PCR primer sets (Bioneer, Daejeon, South Korea) for alkaline phosphatase (ALP), osteocalcin (OC), histone deacetylase 5 (HDAC5), Runt-related transcription factor2 (Runx2), and glyceraldehyde-3-phosphate dehydrogenase (GAPDH; as the housekeeper gene) were used. The expression level of each target gene was normalized to that of the internal GAPDH control and is presented as relative expression. To confirm the constant expression level of the housekeeping gene during total RNA extractions, GAPDH real-time PCR was performed. Real-time PCR was quantified using an AB 7500 instrument (Applied Biosystems) with GAPD (GAPDH) Endogenous Control (FAM/MGB Probe; Primer Limited).

### Western blotting

Cells were washed with ice-cold phosphate-buffered saline (PBS, pH7.2), scraped, and lysed into 20 μl of 1 × RIPA lysis buffer (ROCKLAND, Pennsylvania, United States). The cell suspension was incubated on ice for 30 min and cell debris was pelleted by centrifugation at 12,000 rpm for 20 min at 4 °C. The supernatant containing the protein fraction was collected and boiled for 5 min, then stored at –20 °C until further analysis.

Equal amounts of protein from each treatment group were resolved on a 2%–10% SDS–PAGE gel (70 V, 50 min) and subsequently transferred onto a nitrocellulose membrane (100 V, 40 min). Blots were blocked with 5% bovine serum albumin in a solution of TBS containing 0.1% Tween 20 (TBST) for 1 h prior to incubation with the primary antibody overnight at 4 °C.

Primary antibodies were diluted in TBST solution containing 5% BSA at a ratio of 1:500. After incubation for 5 min each with TBST solution, the blots were incubated for 1 h at room temperature with the appropriate secondary antibody at a 1:1,000 dilution in TBST solution containing 5% BSA. Subsequently, the blots were washed 3 times for 10 min each with TBST solution, and the antibody binding was detected using the Chemi-Image Analysis System (BIORAD, California, United States).

The primary antibodies used here were as follows: anti-alkaline phosphatase (ALP) antibody (sc-365765, Santa Cruz, California, United States), anti-osteocalcin (OC) antibody (sc-74495, Santa Cruz, California, United States), and anti-beta-actin mouse monoclonal antibody (sc-517582, Santa Cruz, California, United States). The secondary antibody used here was the mouse IgG kappa binding protein conjugated to horseradish peroxidase (HRP).

### Statistical analysis

We performed all quantitative experiments at least in triplicate. The data are presented as the mean ± standard deviation. The statistical significance of the in vitro test was determined by two-tailed Student’s *t*-test and one-way analysis of variance (ANOVA) after Bonferroni’s correction using the GraphPad Prism software (5.0; GraphPad, San Diego, CA, USA). Statistical significance was set at *P* < 0.05.

### Ethics approval

This study was performed according to the recommendations of the Guide for the Care of the National Institutes of Health. The protocol was approved by the Use Committee of the Clinical Research Institute, Seoul National University Hospital, Seoul, Korea (permit number: 07–2018-29).

## Results

### In vitro cytotoxicity and penetration of LK peptides

First, we investigated the e cytotoxicity of ac-LK on MSCs. MSCs were treated ac-LK up to 1 μM for 2 days and exhibited almost no cytotoxicity (Fig. 3A). Next, we also examined the cell-penetrating abilities of TAMRA-LK in MSCs on the concentration of 50 nM and 300 nM through flow cytometry. Cells were incubated with TAMRA-LK peptides for 1 day and showed 79.6% of the cells even at a concentration of 50 nM, and they fully penetrated the cells at a concentration of 300 nM (Fig. 3B).

### In vitro cellular uptake of miRNA 2861

**Fig. 2:**
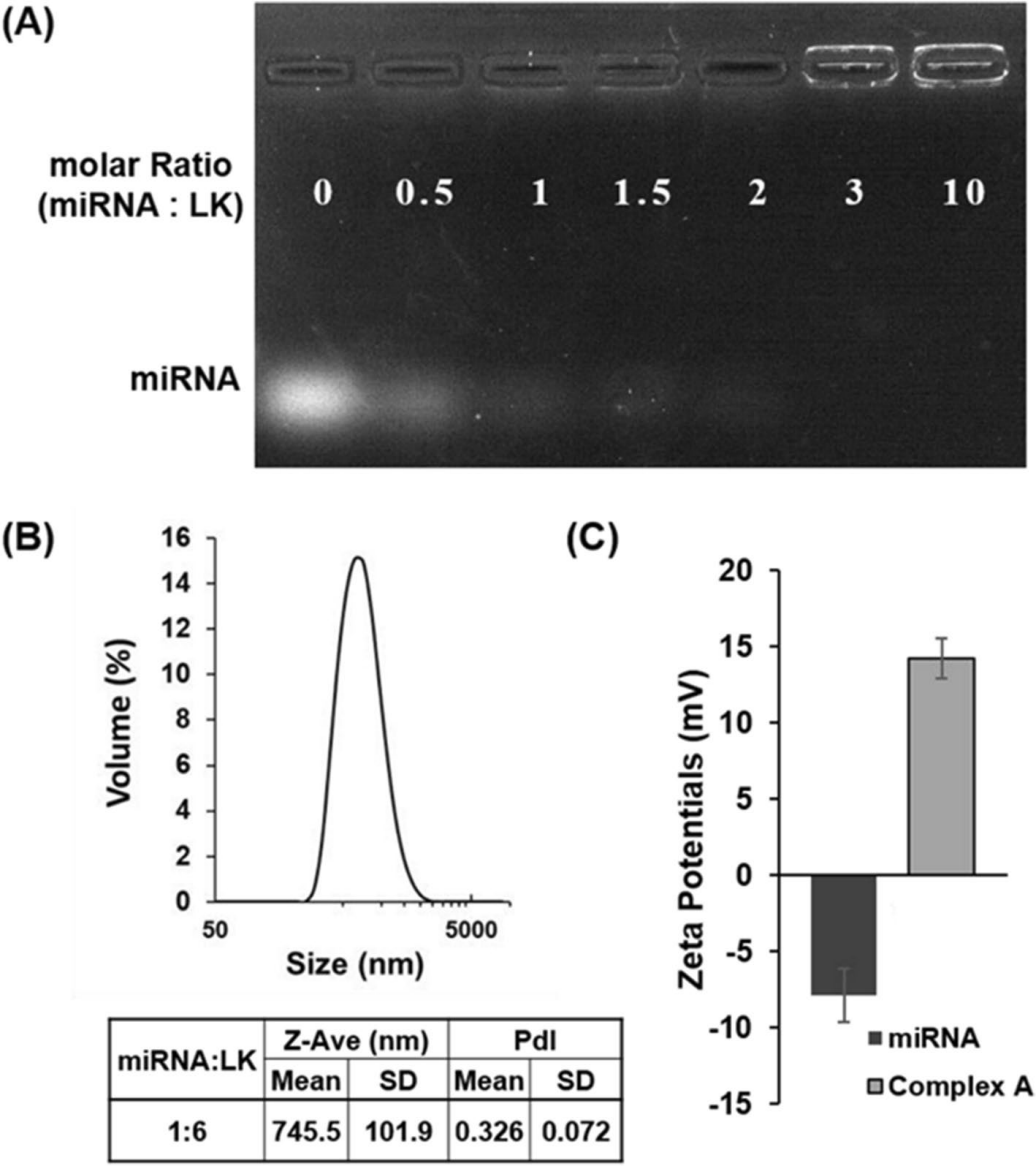
**A** Gel retardation assay of complex of miRNA 2861 and ac-LK. The preformed miRNA/LK complexes were analyzed by electrophoresis on 1% agarose gel. **B** Size analysis of Complex A (50 nM of miRNA 2861 and 300 nM of ac-LK) and **C** zeta potential of miRNA 2861 and Complex A. particle size and charge were examined by DLS using a Zetasizer

**Fig. 3:**
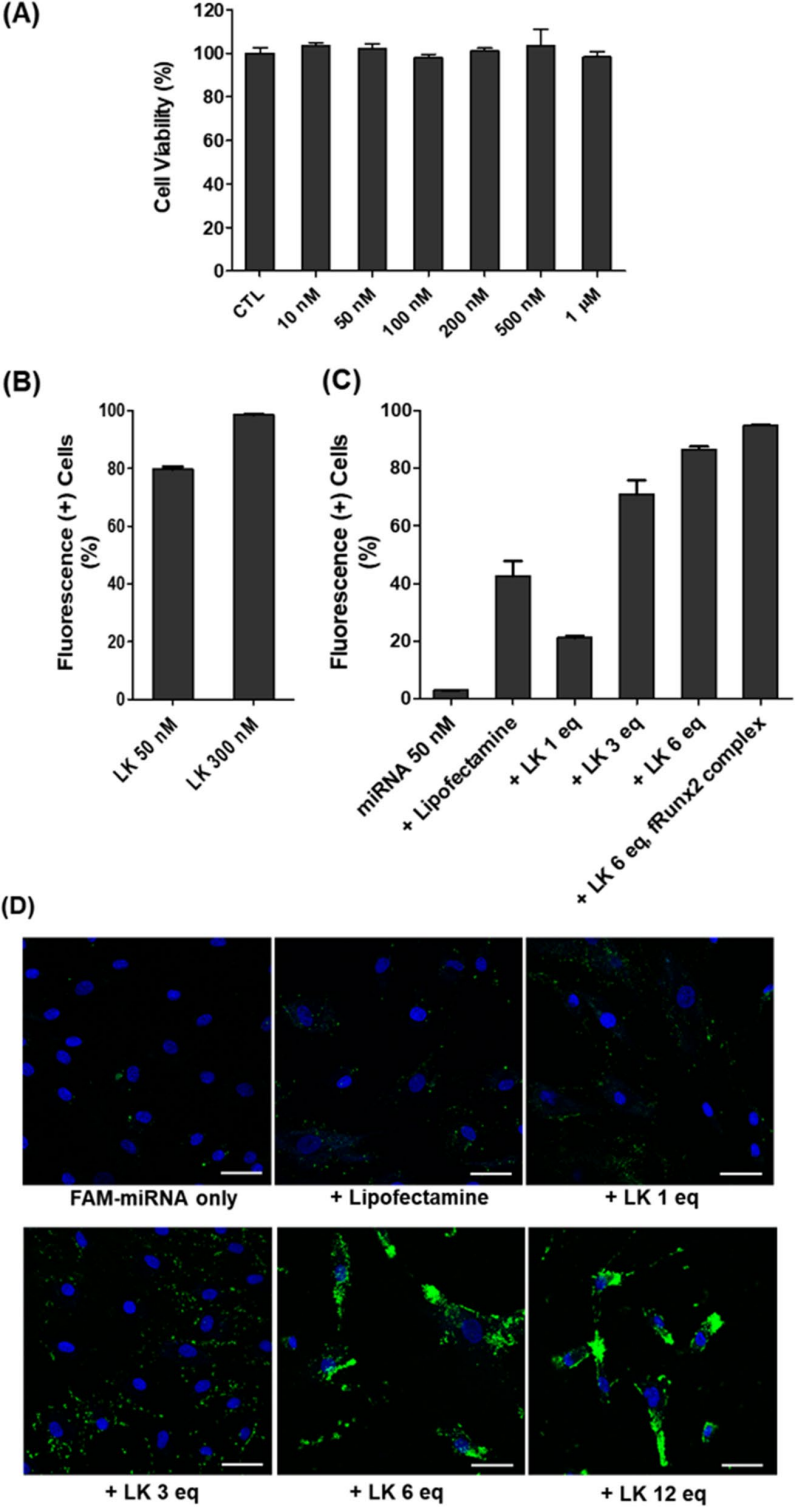
**A** in vitro cytotoxicity of ac-LK on MSCs. **B** Cellular uptake activity of Tamra-LK on MSCs at 50 nM and 300 nM. **C** Cellular uptake activities of FAM-miRNA 2861 only, complexes (50 nM of FAM-miRNA 2861 with 1, 3 and 6 equivalent of ac-LK) using flow cytometry, **D** Representative Confocal laser scanning electron microscopic images of cell treated with FAM-miRNA 2861 only (50 nM) and Complexes (50 nM of FAM-miRNA 2861 with 1, 3, 6 and 12 equivalent ac-LK). The complex of FAM-miRNA 2861 and Lipofectamine was used as a positive control. Fluorescence ( +) cells means the relative fluorescence intensity of FAM-miRNA 2861 based on control cells with no fluorescence

Through electrophoresis, it was confirmed that miRNA 2861 formed particles with ac-LK stably over 3 times the molar ratio to ac-LK. Complex A (miRNA 50 nM and LK 300 nM, Fig. 3A) was 745.5 ± 101.9 nm in size (Fig. 2B) and 14.2 ± 1.32 in charge (Fig. 2C), that ac-LK interacted with the negative charge of miRNA electrostatically and formed a particle. Complex B and Complex C also showed the particle formation with the size of 673.9 ± 170.1 nM and 766.7 ± 54.0 nM respectively (Fig. S1). Next, the morphology of Complex A, Complex B and Complex C were verified using TEM (Fig. S2). Interestingly, nano-sized particles were formed in all complexes by simple mixing, which indicates the stable electrostatic interaction between miRNA 2861 and ac-LK.

We examined the ability of ac-LK as a carrier to deliver FAM-labeled miRNA 2861 (FAM-miRNA 2861) into the cells via the formation of a complex with ac-LK using FACS and CLSM (Fig. 3C and D). miRNA 2861 alone was hardly able to penetrate the cell membrane (2.93% cellular uptake at 50 nM). By contrast, the complexes with ac-LK showed an enhanced cell-penetrating activity compared with FAM-miRNA 2861. A combination of 50 nM FAM-miRNA 2861 and 300 nM ac-LK resulted in 86.5% fluorescence-positive cells. A combination of 50 nM FAM-miRNA 2861 and 600 nM ac-LK resulted in 94.9% fluorescence-positive cells. However, in the lipofectamine group, only 42.6% of cells were positive for fluorescence. Intracellular green fluorescence of FAM-miRNA 2861 was observed using CLSM. Almost no green fluorescence was detected in the FAM-miRNA 2861-only group, whereas the combination group exhibited intense green fluorescence in the cytoplasm.

Next, we investigated the endosomal escape tendency of FAM-miRNA in time dependent after treating with Complex B (50 nM of FAM-miRNA and 600 μM of ac-LK). We observed that the red signal of lysotracker stained late endosome or lysosome overlapped with the green signal of FAM-miRNA 2861 until 12 h, but did not overlap after 24 h and confirmed that FAM-miRNA did some degree of endosomal escape (Fig. S3).

### Assessment of calcium deposition using Alizarin red staining

**Fig. 4:**
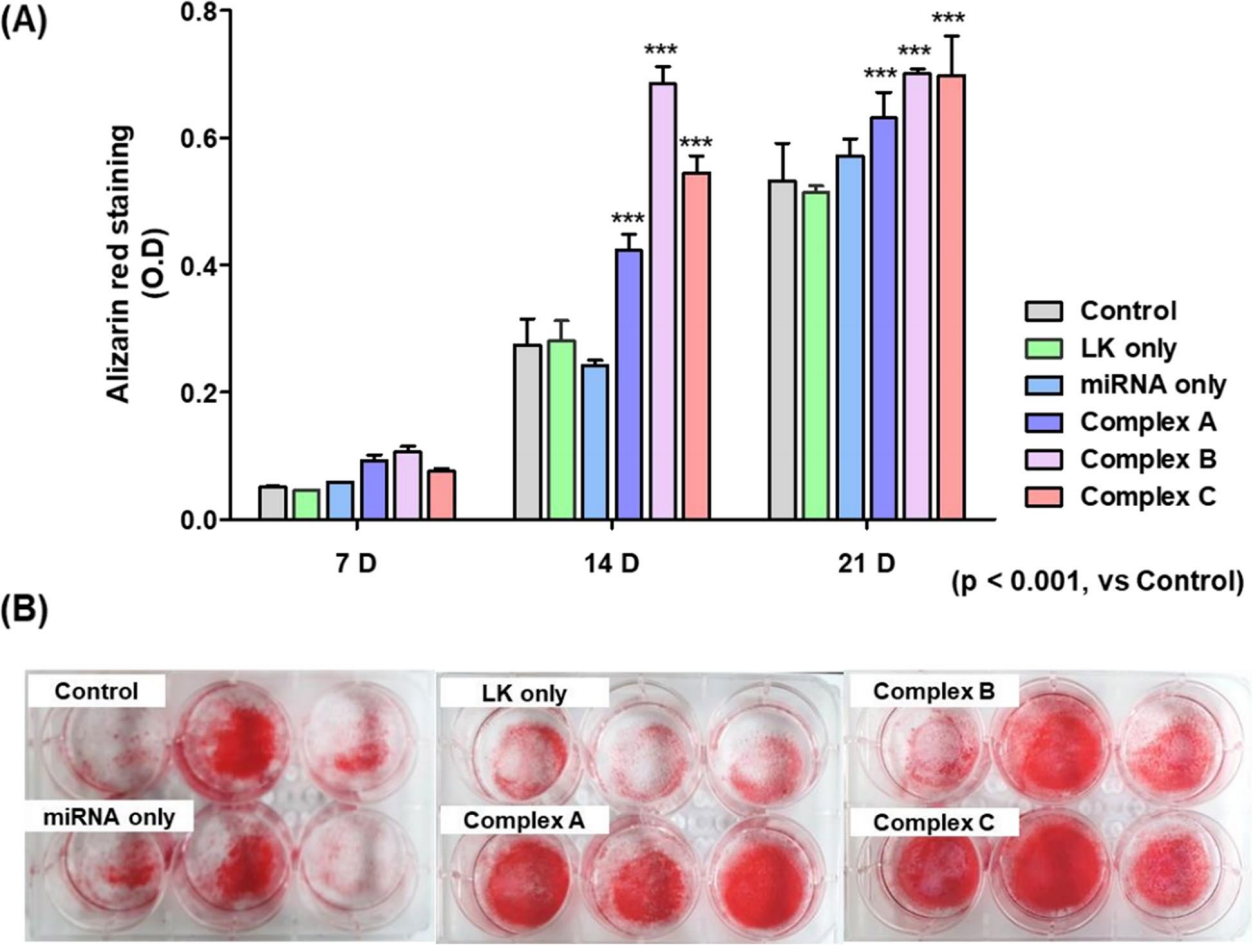
Osteogenic differentiation property of MSCs in vitro. The cells were incubated in the medium containing PBS, ac-LK (800 nM), Complex A (miRNA 2861, 50 nM and ac-LK, 300 nM), Complex B (miRNA 2861, 50 nM and ac-LK, 600 nM) and Complex C (Complex B and fRunx2 100 nM, LK 500 nM) for 24 h. The graphs represent the quantitative analysis of alizarin red staining (**A**) on 7, 14, and 21 days and optical microscopic images on day 21. **B** Representative images of alizarin red staining showed the degree of alizarin red staining for mineral deposition on 21 days

We investigated the osteogenic effect of miRNA 2861 and Complex A, B, and C in MSCs. The differentiation of MSCs into osteoblasts was evaluated by measuring the amount of calcium deposition through alizarin red staining. On days 14 and 21, all complex groups of miRNA 2861 and ac-LK showed significantly more calcium deposition than did the control and miRNA-only group, indicating a tendency toward an increase over time. In particular, the most significant difference was observed at 2 weeks in the complex B group. On day 21, calcium deposition was slightly increased in both the miRNA-only group and the control group, whereas the complex groups still showed a significant increase in intensity (Fig. 4A). Alizarin red staining imaging also confirmed the higher increase in calcium deposition in the complex groups (Figs. 4A and S4-5).

### Gene expression of markers of differentiation

**Fig. 5:**
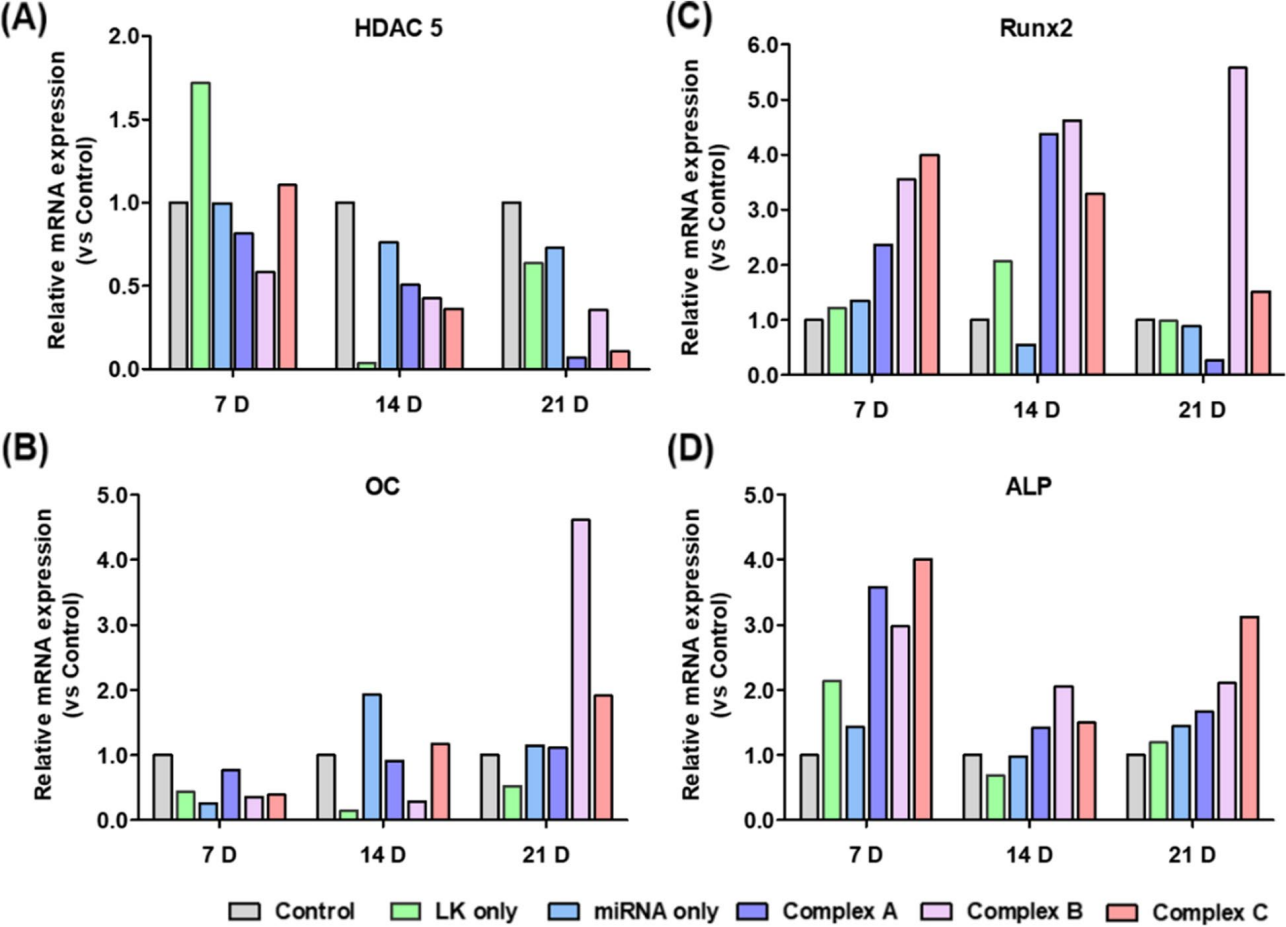
Investigation of gene expression related with osteoblasts differentiation. The cells were incubated in the medium containing PBS, ac-LK (800 nM), Complex A (miRNA 2861, 50 nM and ac-LK, 300 nM), Complex B (miRNA 2861, 50 nM and ac-LK, 600 nM) and Complex C (Complex B and fRunx2 100 nM, LK 500 nM) for 24 h. Then the expression level of HDAC5 (**A**), Runx2 (**B**), OC (**C**) and ALP (**D**) were examined on 7, 14, and 21 days through qRT-PCR. The data represents the relative value compared with PBS, control group. This experiment was performed once

RT-qPCR was used to quantify the increase or decrease in the expression of various marker genes, i.e., *HDAC5*, *OC*, *ALP*, and *Runx2*. *HDAC5* is the primary target of miRNA 2861 ^12^. On days 7, 14, and 21 after seeding, the gene expression level of *HDAC5* in the complex groups was lower than that observed in the control and miRNA 2861-only groups (Fig. 5A). In the case of Runx2 and ALP, which are a major transcription factor and protein, the complex groups exhibited higher expression levels than did the control group and the miRNA 2861-alone group on days 7, 14, and 21 (Fig. 5C, D). Regarding the expression of the *OC* gene, there was no significant difference or trend until the 14^th^ day. On day 21, however, a very high increase was observed in the complex B group compared with the control group (Fig. 5B). Among the results obtained on the 21^st^ day, especially in the complex B group, the expression of *HDAC* was suppressed, and the expression of *ALP*, *OC*, and *Runx2* was increased compared with the control and miRNA 2861-only group (Table S1).

### Protein expression during differentiation

**Fig. 6:**
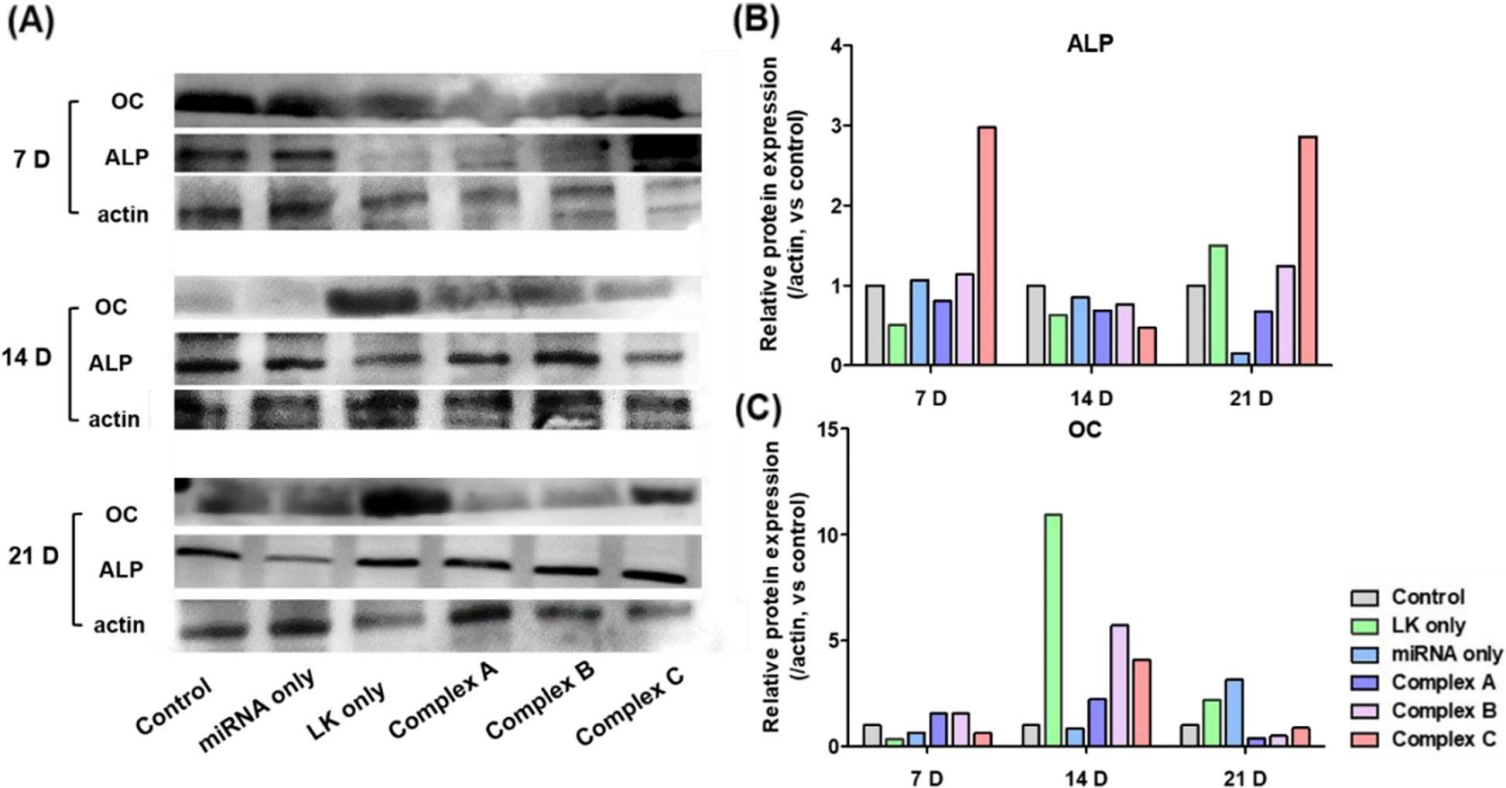
Investigation of protein expression. The cells were incubated in the medium containing PBS, ac-LK (800 nM), Complex A (miRNA 2861, 50 nM and ac-LK, 300 nM), Complex B (miRNA 2861, 50 nM and ac-LK, 600 nM) and Complex C (Complex B and fRunx2 100 nM, ac-LK 500 nM) for 24 h. The expression level of ALP (**B**) and OC (**C**) were examined at 4, 7, 14, and 21 days through western blot. The data represents the relative value compared with PBS, control group. Quantities value was analyzed using the Prizm 5.0 software. This experiment was performed once

Western blotting was performed to confirm that the expression levels of the OC and ALP proteins were similar to the RNA level patterns (Fig. 6A). Although the LK-only group exhibited a high ALP and OC expression level, the expression of the ALP protein was higher in the complex groups compared with the control and miRNA-only groups on day 21 (Fig. 6B). With the exception of the LK-only group, the OC expression levels in the complex groups were high compared with those detected in the control and miRNA-only groups (Fig. 6C). In the complex groups with the combination of miRNA 2861 and ac-LK, the mRNA expression levels of ALP on 7, 14, and 21 days were higher in than those in other groups. The level of OC on 21 days was higher compared to other groups. However, the protein expression of ALP and OC in the complex groups were higher on 7 and 21 days and 14 days compared to other groups, respectively. It means protein expression may not always be followed after corresponding gene expression.

## Discussion

In this study, we showed that the efficient delivery of miRNA 2861 using LK increased the positive effects observed on osteoblast differentiation. The mineralization detected in the complex groups was enhanced compared with the miRNA-only and the control group. The entry of miRNA 2861 into cells augmented the expression of the Runx2, OC, and ALP genes by inhibiting the expression of the HDAC5 gene, and had a powerful effect on osteoblast differentiation in human MSCs in the present study.

Bone remodeling is important for the maintenance of proper bone structure; in turn, osteoporosis and fragile fractures are the result of dysfunctional bone remodeling [10, 18]. Epigenetics usually alters gene expression in response to external and internal environmental signals, including aging. Therefore, the cause of osteoporosis-induced bone dysregulation is more likely attributable to the interaction between the genetic code and the environment [10, 19]. Previous studies reported that histone modification and miRNAs modulated bone remodeling, including osteoblast differentiation [20, 21]. Among 18 human HDACs, it was reported that an increase in HDAC5 was associated with low expression of Runx2, a master transcription factor of osteoblast differentiation from MSCs [15]. In addition, miRNA 2861 promoted osteoblast differentiation by targeting HDAC5 and increasing Runx2 protein production in mice [15, 21]. In our study, we showed the decreased expression of HDAC5 and increased expression of Runx2 after penetration of miRNA 2861 into human MSCs. Therefore, miRNA 2861 may be a good therapeutic agent for promoting osteoblast differentiation.

In this study, the effect of miRNA 2861 on human MSCs was examined, and LK peptides were used to increase the cellular uptake of miRNA 2861. We had reported previously that the LK peptide and its derivatives were effective in delivering small drugs, proteins, nanoparticles, etc. into cells [22–24]. The cellular uptake of FAM-miRNA started to increase when using 3 or more equivalents of ac-LK, and was optimized at 6 equivalent amount of ac-LK and slightly increased with 12 equivalent amount of LK (Complex B). Therefore, we designated the experimental groups as Complex A and B for investigation osteogenic differentiation. Besides, in our previous research, we examined the complex of fRunx2 and LK peptides on osteogenic differentiation and obtained the results that the complex of fRunx2 and LK peptide increased osteogenic differentiation [16]. Therefore, we prepared the Complex C mixing Complex B and Complex of fRunx2 and ac-LK to investigate the synergistic effect of miRNA and fRunx2.

In human MSCs, the miRNA 2861–LK complex groups (complex A–C groups) alone clearly showed upregulation of the Runx2, OC, and ALP genes via the inhibition of the expression of the HDAC5 gene. MSCs are multipotent stromal cells and the source of osteoblasts, chondrocytes, and adipocytes [12, 25]. In the alizarin red staining and Western blotting studies, the miRNA 2861–LK complex showed increased mineralization and the production of proteins related to mineralization.

Therefore, differentiation of MSCs into osteoblasts is an attractive treatment method for skeletal regeneration. Various osteogenic factors, including miRNAs, are involved in the differentiation of MSCs into osteoblasts [13, 15]. Because elderly patients in need of surgical treatment related to bone formation have a decreased capability of bone remodeling and a weak skeletal environment, the promotion of osteoinduction may be beneficial in technologies of bone substitution [2]. miRNA 2861 could selectively upregulate osteogenesis without stimulating adipogenesis, chondrogenesis, and myogenesis. Therefore, based on previous studies and the present investigation, the increase in the penetration of miRNA 2861 using LK peptides into human MSCs may be one an approach to enhance bone formation.

This study had several limitations. We did not assess whether miRNA 2861 had an effect on the non-osteoblast lineage of human MSCs and did not investigate the effect of miRNA 2861 on other signal pathways related to osteoblast differentiation. Future studies are needed to assess the effect on the non-osteoblast lineage and other signaling pathways. In addition, we used human MSCs derived from young adults, rather than elderly people. Therefore, it is unclear whether the miRNA 2861–LK complex has a positive effect on aging MSCs. Through additional studies, we will reveal the augmentation of osteoblast differentiation in MSCs derived from the bone marrow of elderly patients. We performed some different experiments using LK before. The LK alone may influenced on gene expression compared to control group sometimes in the previous experiments [16]. In Fig. 6, we showed the highest expression of OC and ALP was found in the group treated only with LK. However, we cannot explain the reason why LK alone may have effect on some specific gene expression not yet. Therefore, we have a future study to investigate the effect of LK on gene expression. In the present study, we did not show the in vivo effect of miRNA 2861 using bone architecture. We would plan the future study to verify the change of bone architecture at the in vivo level after administration of miRNA 2861.

A future investigation using animal models, such as the bone fusion model or fracture healing model, is also needed. There are many miRNAs such as miRNA 449a and 29b involved in osteogenic differentiation. Therefore, future studies will need to use various combinations of miRNAs with CPP to study their effects on osteogenesis.

## Conclusion

The LK peptide effectively delivered miRNA 2861 into the cytoplasm of human MSCs. As a result, the complex of miRNA 2861 with LK may have a positive effect on the osteogenic differentiation from MSCs and mineralization. Therefore, a therapy using miRNA 2861 combined with LK may be a good therapeutic candidate for osteogenesis.

## Supplementary Information

**Additional file 1: Table S1.**This table presented raw analysis data to investigate gene expression related with osteoblasts differentiation (figure 5).

**Additional file 2: Figure S1.** (A) Size analysis of complex A, complex B and complex C (C) Representative table of size results. (C) Zeta potential of miRNA 2861, LK, Complex A, Complex B and Complex C. The complex A and B were prepared using 500 nM miRNA 2861 and 6, and 12 molar equivalent amounts of LK. The Complex C was manufactured by mixing 500 nM of fRunx2 and 1μm of ac-LK with the complex B. Particle size and charge were examined by DLS using a Zetasizer. All experiments were triplicated.

**Additional file 3: Figure S2.** TEM images of miRNA 2861 (a), LK (b), complex A (c), complex B (d) and complex C. The complex A and B were prepared using 2 μM miRNA 2861 and 6, and 12 equivalent amounts of ac-LK. The Complex C was manufactured by mixing 2 μM of fRunx2 and 10 μM of ac-LK with the complex B. Scale bar indicates 500 nm.

**Additional file 4: Figure S3.** Representative merged images of time dependent cellular uptake activity and endosomal escape ability of FAM-miRNA 2861 of Complex B (50 nM of FAM-miRNA 2861 and 600 nM of LK). Cells were incubated with FAM-miRNA 2861 and Complex B for 2h, 4 h, 6 h, 12 h and 24 h individually and analyzed by confocal laser scanning electron microscope. Scale bar indicates 20 μm. Colors represent green of FAM-miRNA 2861, red of late endosome or lysosome stained by Lysotracker, and blue of nucleus stained by Hoechst 33342).

**Additional file 5: Figure S4.** Representative images of Alizarin red staining. The cells were incubated in the medium containing PBS, ac-LK (800 nM), Complex A (miRNA 2861, 50 nM and ac-LK, 300 nM), Complex B (miRNA 2861, 50 nM and ac-LK, 600 nM) and Complex C (Complex A and fRunx2 100nM, LK 500 nM) for 24h. The degree of alizarin red staining for mineral deposition were investigated at 7, 14, and 21 days.

**Additional file 6: Figure S5.** Representative optical microscopic images of Alizarin red staining. The cells were incubated in the medium containing PBS, ac-LK (800 nM), Complex A (miRNA 2861, 50 nM and ac-LK, 300 nM), , Complex B (miRNA 2861, 50 nM and ac-LK, 600 nM) and Complex C (Complex A and fRunx2 100nM, LK 500 nM) for 24h. The degree of alizarin red staining for mineral deposition were investigated at 7, 14, and 21 days.

## Data Availability

All data is available upon request to the corresponding author.

## Abbreviations

MSCs: mesenchymal stem cells
BMP: bone morphogenetic protein
HDACs: histone acetylase inhibitors
miRNAs: micro RNAs
CPPs: cell-penetrating peptides
LK: leucine and lysine residues
PYBOP: benzotriazole-1-yl-oxy-tris-pyrrolidino-phosphonium hexafluorophosphate
DMF: Dimethylformamide
DCM: 1,2-dichloromethane
DIPEA: N,N-diisopropylethylamine
TFA: trifluoroacetic acid
TIS: triisopropylsilane
TES: triethylsilane
TAMRA: piperidine, and 5(6)-carboxytetramethylrhodamine
OM: osteogenic medium
ALP: alkaline phosphatase
OC: osteocalcin
GAPDH: glyceraldehyde-3-phosphate dehydrogenase
Runx2: Runt-related transcription factor2
PBS: phosphate-buffered saline
ANOVA: one-way analysis of variance
