# Supplementary material for: The complex of miRNA2861 and cell-penetrating, dimeric α-helical peptide accelerates the osteogenesis of mesenchymal stem cells
Source: Biomater Res. 2022 Dec 29;26:90. doi: 10.1186/s40824-022-00336-9 (PMC9798695; doi:10.1186/s40824-022-00336-9)
Supplement: Supplementary file 1 — Additional file 1: Table S1.This table presented raw analysis data to investigate gene expression related with osteoblasts differentiation (figure 5). [file 40824_2022_336_MOESM1_ESM.docx]

Table S1.

|  | 7 days | | | | 14 days | | | | 21 days | | | |
| --- | --- | --- | --- | --- | --- | --- | --- | --- | --- | --- | --- | --- |
|  | HDAC5 | Runx2 | OC | ALP | HDAC5 | Runx2 | OC | ALP | HDAC5 | Runx2 | OC | ALP |
| Control | 1 | 1 | 1 | 1 | 1 | 1 | 1 | 1 | 1 | 1 | 1 | 1 |
| LK only | 0.99 | 1.36 | 0.26 | 1.44 | 0.76 | 0.55 | 1.93 | 0.98 | 0.73 | 0.89 | 1.14 | 1.44 |
| miRNA only | 1.72 | 1.22 | 0.44 | 2.14 | 0.04 | 2.06 | 0.15 | 0.68 | 0.64 | 0.98 | 0.53 | 1.20 |
| Complex A | 0.82 | 2.37 | 0.77 | 3.58 | 0.51 | 4.38 | 0.91 | 1.41 | 0.07 | 0.27 | 1.11 | 1.66 |
| Complex B | 0.58 | 3.56 | 0.36 | 2.98 | 0.43 | 4.63 | 0.29 | 2.05 | 0.35 | 5.58 | 4.61 | 2.11 |
| Complex C | 1.11 | 4.00 | 0.39 | 4.00 | 0.36 | 3.29 | 1.16 | 1.49 | 0.11 | 1.52 | 1.91 | 3.12 |

This table presented raw analysis data to investigate gene expression related with osteoblasts differentiation (figure 5).
