# Supplementary figures and images for: The complex of miRNA2861 and cell-penetrating, dimeric α-helical peptide accelerates the osteogenesis of mesenchymal stem cells
Source: Biomater Res. 2022 Dec 29;26:90. doi: 10.1186/s40824-022-00336-9 (PMC9798695; doi:10.1186/s40824-022-00336-9)

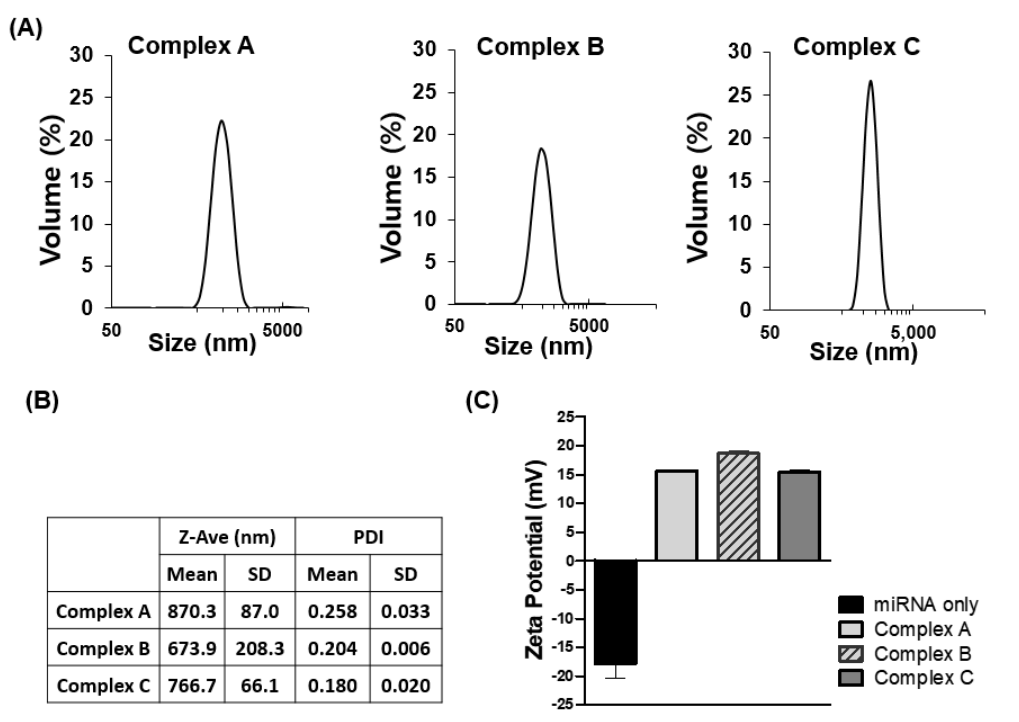

Supplement: Supplementary file 2 — Additional file 2: Figure S1. (A) Size analysis of complex A, complex B and complex C (C) Representative table of size results. (C) Zeta potential of miRNA 2861, LK, Complex A, Complex B and Complex C. The complex A and B were prepared using 500 nM miRNA 2861 and 6, and 12 molar equivalent amounts of LK. The Complex C was manufactured by mixing 500 nM of fRunx2 and 1μm of ac-LK with the complex B. Particle size and charge were examined by DLS using a Zetasizer. All experiments were triplicated. [file 40824_2022_336_MOESM2_ESM.jpg]

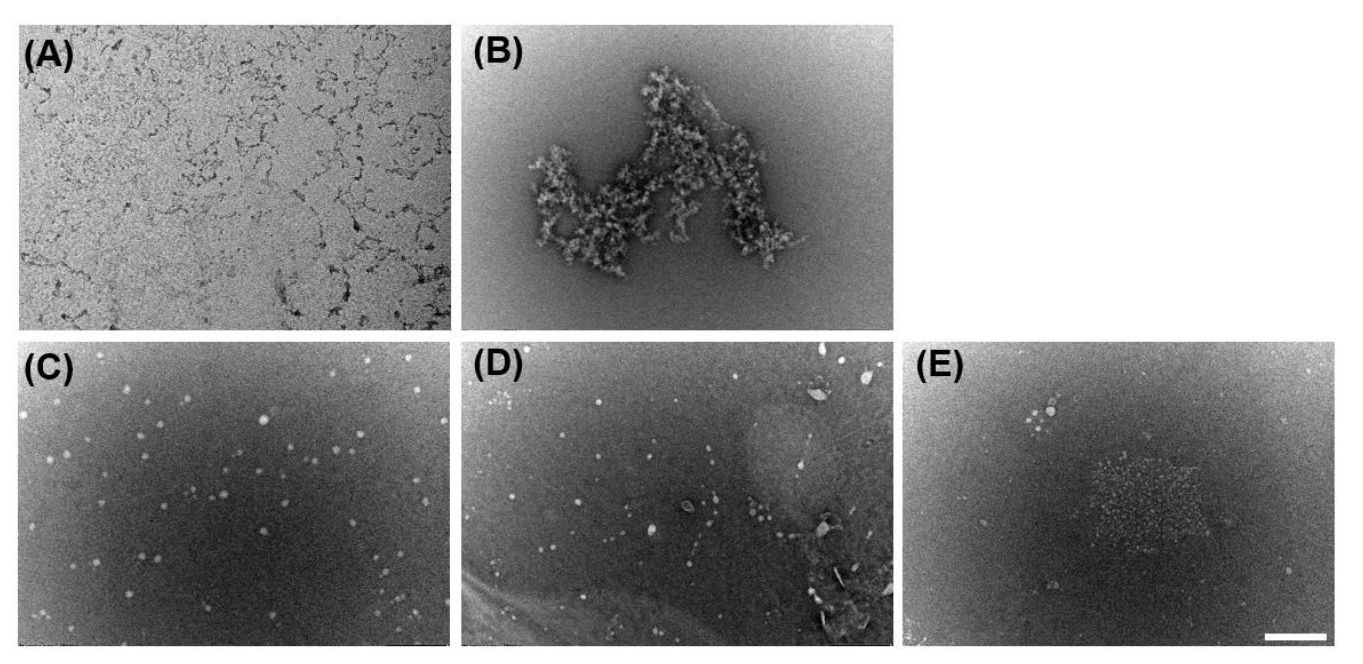

Supplement: Supplementary file 3 — Additional file 3: Figure S2. TEM images of miRNA 2861 (a), LK (b), complex A (c), complex B (d) and complex C. The complex A and B were prepared using 2 μM miRNA 2861 and 6, and 12 equivalent amounts of ac-LK. The Complex C was manufactured by mixing 2 μM of fRunx2 and 10 μM of ac-LK with the complex B. Scale bar indicates 500 nm. [file 40824_2022_336_MOESM3_ESM.jpg]

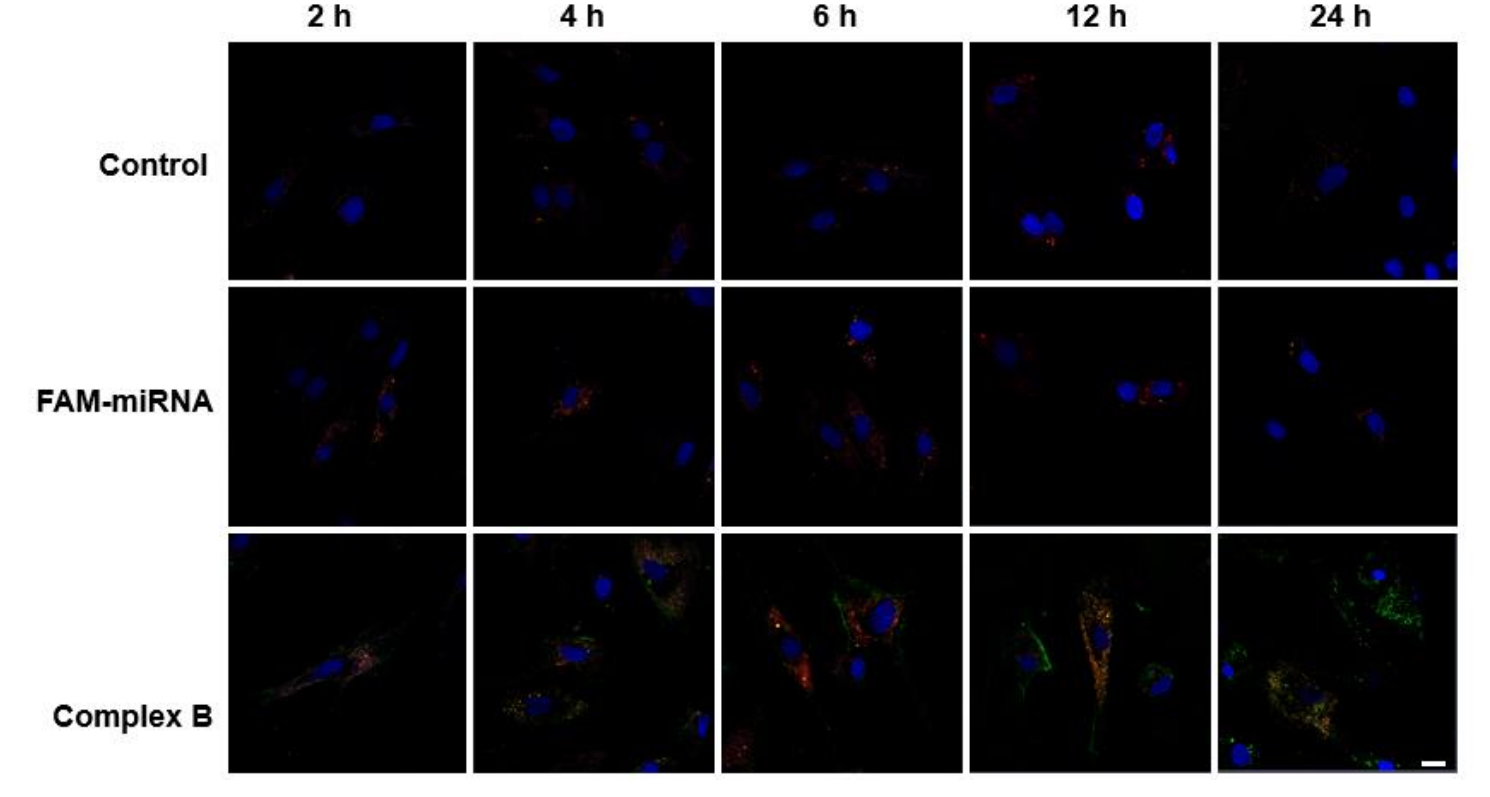

Supplement: Supplementary file 4 — Additional file 4: Figure S3. Representative merged images of time dependent cellular uptake activity and endosomal escape ability of FAM-miRNA 2861 of Complex B (50 nM of FAM-miRNA 2861 and 600 nM of LK). Cells were incubated with FAM-miRNA 2861 and Complex B for 2h, 4 h, 6 h, 12 h and 24 h individually and analyzed by confocal laser scanning electron microscope. Scale bar indicates 20 μm. Colors represent green of FAM-miRNA 2861, red of late endosome or lysosome stained by Lysotracker, and blue of nucleus stained by Hoechst 33342). [file 40824_2022_336_MOESM4_ESM.jpg]

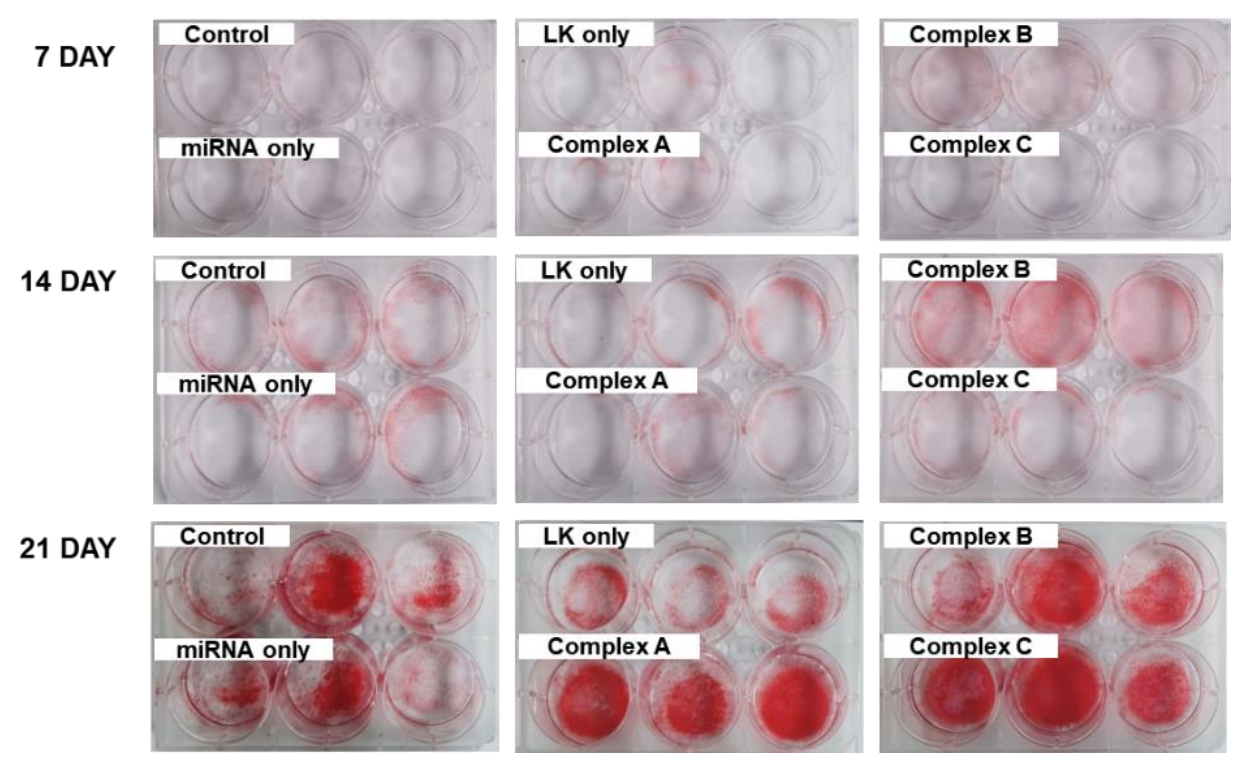

Supplement: Supplementary file 5 — Additional file 5: Figure S4. Representative images of Alizarin red staining. The cells were incubated in the medium containing PBS, ac-LK (800 nM), Complex A (miRNA 2861, 50 nM and ac-LK, 300 nM), Complex B (miRNA 2861, 50 nM and ac-LK, 600 nM) and Complex C (Complex A and fRunx2 100nM, LK 500 nM) for 24h. The degree of alizarin red staining for mineral deposition were investigated at 7, 14, and 21 days. [file 40824_2022_336_MOESM5_ESM.jpg]

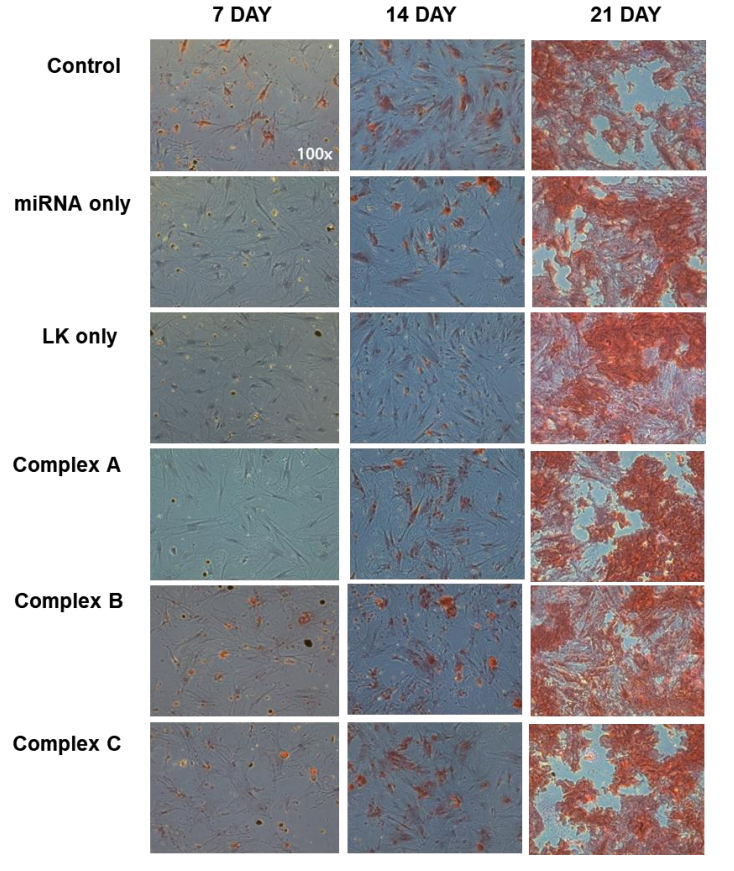

Supplement: Supplementary file 6 — Additional file 6: Figure S5. Representative optical microscopic images of Alizarin red staining. The cells were incubated in the medium containing PBS, ac-LK (800 nM), Complex A (miRNA 2861, 50 nM and ac-LK, 300 nM), , Complex B (miRNA 2861, 50 nM and ac-LK, 600 nM) and Complex C (Complex A and fRunx2 100nM, LK 500 nM) for 24h. The degree of alizarin red staining for mineral deposition were investigated at 7, 14, and 21 days. [file 40824_2022_336_MOESM6_ESM.jpg]
